# Oral Gastrografin Facilitates Delineation of Intestinal Tracts in CT-Based Brachytherapy for Uterine Cervical Cancer

**DOI:** 10.7759/cureus.8367

**Published:** 2020-05-30

**Authors:** Daisuke Irie, Kazutoshi Murata, Takuya Kaminuma, Takahiro Oike, Tatsuya Ohno

**Affiliations:** 1 Department of Radiation Oncology, Gunma University Graduate School of Medicine, Maebashi, JPN; 2 Heavy Ion Medical Center, Gunma University, Maebashi, JPN

**Keywords:** uterine cervical cancer, radiation therapy, brachytherapy, computed tomography, oral contrast, gastrografin, diatrizoate meglumine, diatrizoate sodium

## Abstract

Three-dimensional image-guided brachytherapy (3D-IGBT) using computed tomography (CT) is an essential component of definitive radiation therapy for uterine cervical cancer (UCC). Treatment planning for CT-based 3D-IGBT requires delineating the high-risk clinical target volume (CTV_HR_) and the organs at risk (OARs), which is difficult when the small intestine is adjacent to those delineation targets. Uncertainty in target delineation threatens the validity of 3D-IGBT treatment plans. To address this issue, we introduce the use of diatrizoate meglumine and diatrizoate sodium (gastrografin), an orally administrable iodine-based radiopaque contrast agent. We present two cases of UCC treated with CT-based 3D-IGBT and describe how intraluminal enhancement of the small intestine by oral gastrografin pretreatment facilitated discrimination between the small intestine and the adjacent CTV_HR_ (case no.1) or the rectosigmoid colon (case no. 2). Oral gastrografin pretreatment is a simple and cost-effective method that allows distinguishing the small intestine from the adjacent delineation target (i.e., CTV_HR_ and the OARs) in CT-based 3D-IGBT for UCC.

## Introduction

Uterine cervical cancer (UCC) causes more than 300,000 deaths worldwide annually, and its mortality ranks fourth among all cancers [1]. Concurrent chemoradiotherapy is the standard definitive treatment for locally advanced UCC [2,3]. The standard protocol for definitive radiotherapy consists of a combination of external beam radiation therapy (EBRT) to the pelvis and brachytherapy to the primary tumor [4]. For the latter, the use of three-dimensional image-guided brachytherapy (3D-IGBT) is becoming widespread. Although magnetic resonance is the gold standard imaging modality for 3D-IGBT, computed tomography (CT)-based 3D-IGBT is prevalent in clinical practice [5,6]. In the treatment planning of CT-based 3D-IGBT, dose prescription is performed based on the 3D-reconstructed volumes of the high-risk clinical target volume (CTV_HR_) and those of the organs at risk (OARs), such as the sigmoid colon, the rectum, and the bladder [7,8]. CT-based 3D-IGBT achieves favorable tumor local control with minimal adverse effects on OARs [8,9].

During the treatment planning of CT-based 3D-IGBT, delineating the CTV_HR_ or OARs is difficult in cases in which the small intestine is adjacent to those delineation targets because the soft tissues of the intestinal tract and those of the uterus show similar CT values. In such cases, uncertainty regarding the border between the small intestine and the CTV_HR_ or OARs contributes to over- or under-estimation of the dose prescribed to the target volumes. This critically threatens tumor local control or OAR tolerability because brachytherapy shows a steep dose fall-off. In this case report, we address this issue by introducing the use of diatrizoate meglumine and diatrizoate sodium (gastrografin), an orally administrable iodine-based radiopaque contrast agent. Oral gastrografin pretreatment is a simple and cost-effective method that helps practitioners to distinguish the small intestine from the adjacent delineation target (i.e., CTV_HR_ and the OARs). We present two representative cases, cases no. 1 and no. 2, and show enhanced images of the small intestine adjacent to the CTV_HR_ and to the rectosigmoid colon, respectively.

## Case presentation

Case no. 1

**Figure 1:**
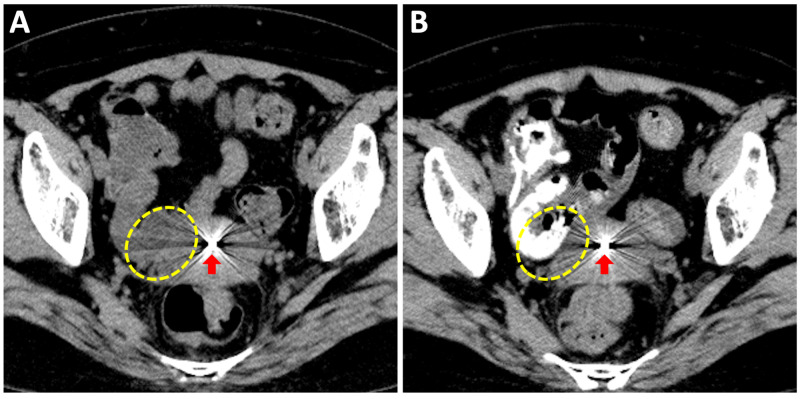
CT images used for treatment planning of brachytherapy obtained in the absence (A) or presence (B) of oral gastrografin pretreatment. The dashed lines show the border regions of the small intestine and the high-risk clinical target volume. The arrow shows the tandem applicator inserted in the endometrial cavity.

A 46-year-old patient with stage IB1 UCC (according to the 2009 definition by the International Federation of Gynecology and Obstetrics [FIGO]) received definitive radiation therapy consisting of EBRT to the pelvis at a dose of 50 Gy in 25 fractions (the latter 30 Gy was delivered using a central shield technique) and four sessions of 3D-IGBT. The CT images obtained at the first IGBT session showed that part of the small intestine was located adjacent to the right anterior portion of the CTV_HR_, and delineating the borders was difficult (Figure 1A). To aid in CTV_HR_ delineation, gastrografin was administered orally four hours before the second IGBT session. In the treatment planning CT, the small intestine was visualized by intraluminal enhancement, which enabled delineation of the CTV_HR_ (Figure 1B).

Case no. 2

**Figure 2:**
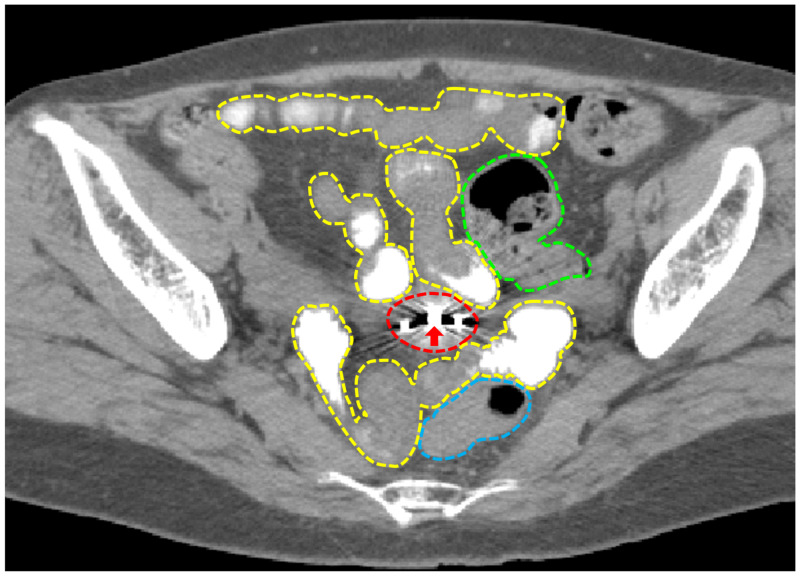
CT images used for treatment planning of brachytherapy with oral gastrografin pretreatment. The dashed yellow, green, blue, and red lines show the small intestine, the sigmoid colon, the rectum, and the high-risk clinical target volume, respectively. The arrow shows the tandem applicator inserted in the endometrial cavity.

A 66-year-old patient with FIGO stage IIA1 UCC received definitive radiation therapy consisting of EBRT to the pelvis at a dose of 50 Gy in 25 fractions (the latter 20 Gy was delivered using a central shield technique) and four sessions of 3D-IGBT. The treatment planning CT for IGBT obtained three hours after oral gastrografin administration showed a cluster of multiple digestive tracts; intraluminal enhancement of the small intestine allowed distinction of this organ from the sigmoid colon and the rectum (Figure 2). The gastrografin pretreatment also helped clarify the CTV_HR_ outline (Figure 2).

## Discussion

Gastrografin is a water-soluble iodine-based radiopaque contrast agent that is widely used for diagnostic purposes. For CT imaging, the agent is diluted at 1:30-50 with water, and approximately 250 ml of diluted solution is administered orally. Based on our clinical experience, the gastrografin solution reaches the small intestine, but not the rectosigmoid colon, in approximately two to four hours after administration, enabling discrimination of the two organs as demonstrated in case no. 2. Of note, there was no specific reason for the difference in the time between the gastrografin pretreatment and CT examination (i.e., four and three hours for case no. 1 and case no. 2, respectively). Gastrografin pretreatment was performed two hours before the initiation of brachytherapy in both cases, followed by the application and packing that took two and one hours for the case no. 1 and no. 2, respectively, contributing to the difference. The use of oral gastrografin in the planning of EBRT for UCC has been reported previously [10,11]. However, to the best of our knowledge, this is the first study to report the use of oral gastrografin for target delineation in CT-based 3D-IGBT and to demonstrate its beneficial effects in representative cases. This method may be particularly useful in 3D-IGBT for bulky and irregularly shaped tumors, which require interstitial needles in addition to the conventional tandem and ovoid applicators to achieve a sufficient dose coverage [12,13]. This is because a single gastrografin pretreatment is effective for multiple CT scans, which are required for IGBT in these cases.

The common side effects of gastrografin include diarrhea, vomiting, and nausea [14]. Oral gastrografin is relatively safe compared with intravenous iodine-based contrast agents; i.e., the contraindication for the former is iodine allergy, whereas that for the latter being severe thyroid, heart, liver, or renal dysfunction, and asthma. In addition, the pharmaceutical cost of gastrografin in Japan was 14.1 JPY/ml in 2019. Together, oral gastrografin pretreatment is a simple and cost-effective option to distinguish the intestine from the adjacent delineation target (i.e., CTV_HR_ and the OARs) in CT-based 3D-IGBT for UCC.

Further research is warranted to elucidate the following points: (i) the effect of the oral gastrografin pretreatment on the shortening of the operation time for IGBT; (ii) the pretreatment timing to obtain intraluminal enhancement of the rectosigmoid colon, and (iii) the safety and efficacy of the pretreatment in combination with spinal anesthesia.

## Conclusions

CT-based 3D-IGBT is an essential component of definitive radiation therapy for UCC. In the treatment planning, delineation of CTV_HR_ and OARs is difficult when the small intestine is adjacent to those delineation targets. Oral gastrografin is a simple and cost-effective pretreatment option that assists in the identification of the border between the small intestine and the adjacent CTV_HR_ or the rectosigmoid colon.

## References

[1] Bray F, Ferlay J, Soerjomataram I, Siegel RL, Torre LA, Jemal A (2018). Global cancer statistics 2018: GLOBOCAN estimates of incidence and mortality worldwide for 36 cancers in 185 countries. CA Cancer J Clin.

[2] Miyasaka Y, Yoshimoto Y, Murata K (2020). Treatment outcomes of patients with adenocarcinoma of the uterine cervix after definitive radiotherapy and the prognostic impact of tumor-infiltrating CD8+ lymphocytes in pre-treatment biopsy specimens: a multi-institutional retrospective study. J Radiat Res.

[3] Marth C, Landoni F, Mahner S (2017). Cervical cancer: ESMO Clinical Practice Guidelines for diagnosis, treatment and follow-up. Ann Oncol.

[4] Monk BJ, Tewari KS, Koh WJ (2007). Multimodality therapy for locally advanced cervical carcinoma: state of the art and future directions. J Clin Oncol.

[5] Grover S, Harkenrider MM, Cho LP (2016). Image guided cervical brachytherapy: 2014 survey of the American Brachytherapy Society. Int J Radiat Oncol Biol Phys.

[6] Tan LT (2011). Implementation of image-guided brachytherapy for cervix cancer in the UK: progress update. Clin Oncol.

[7] Ohno T, Wakatsuki M, Toita T (2017). Recommendations for high-risk clinical target volume definition with computed tomography for three-dimensional image-guided brachytherapy in cervical cancer patients. J Radiat Res.

[8] Zolciak-Siwinska A, Gruszczynska E, Bijok M (2016). Computed tomography-planned high-dose-rate brachytherapy for treating uterine cervical cancer. Int J Radiat Oncol Biol Phys.

[9] Okazaki S, Murata K, Noda SE (2019). Dose-volume parameters and local tumor control in cervical cancer treated with central-shielding external-beam radiotherapy and CT-based image-guided brachytherapy. J Radiat Res.

[10] Banerjee R, Kamrava M (2014). Brachytherapy in the treatment of cervical cancer: a review. Int J Womens Health.

[11] Deodato F, Macchia G, Grimaldi L (2009). Stereotactic radiotherapy in recurrent gynecological cancer: a case series. Oncol Rep.

[12] Wakatsuki M, Ohno T, Yoshida D (2011). Intracavitary combined with CT-guided interstitial brachytherapy for locally advanced uterine cervical cancer: introduction of the technique and a case presentation. J Radiat Res.

[13] Oike T, Ohno T, Noda SE (2014). Can combined intracavitary/interstitial approach be an alternative to interstitial brachytherapy with the Martinez Universal Perineal Interstitial Template (MUPIT) in computed tomography-guided adaptive brachytherapy for bulky and/or irregularly shaped gynecological tumors?. Radiat Oncol.

[14] Farid M, Fikry A, El Nakeeb A (2010). Clinical impacts of oral gastrografin follow-through in adhesive small bowel obstruction (SBO). J Surg Res.

